# A Monograph on Ovarian Tumors

**Published:** 1857-03

**Authors:** T. M. Tweed

**Affiliations:** Eckmansville, Ohio


### SELECTIONS.

*A Monograph on Ovarian Tumors; with an extended view of Ovariotomy as a means of cure.* By T. M. Tweed, M. D., of Eckmansville, Ohio.

There are, perhaps, no organs in the human body in which *cysts* containing fluid are so frequently found developed as in the appendages of the uterus, and particularly in the Ovaria. These sacs or cysts, which have not unfrequently been confounded with hydatids, constitute the disease termed *Ovarian* or *Encysted Dropsy;* and it scarcely admits of a doubt, from the progressive enlargement observed in the Graafian vesicles, that these cysts often originate in a morbid distension of these bodies. In other cases, Ovarian Dropsy arises from the development of a solitary serous cyst in the neighborhood of the uterus, in the folds of the broad ligament, or connected with the Ovaria, if not imbedded in their substance. The whole substance of the Ovary is converted into a large bag containing a fluid, or into a congeries of cysts of different sizes, which have no communication with each other.

These cysts, which differ considerably in the density of their coats, contain fluids which vary in color and consistence. In some it is serous, mixed with a slimy, ropy fluid, like jelly; in others it is a purulent fluid, or dark colored like coffee grounds; in some rare instances the contained matter resembles custard or soft cheese; in others a thick dark brown fluid like treacle is observed.

Cysts containing a fatty, or sebaceous matter, intermingled with hair and teeth and bones, have been frequently met with, either in the substance of one of the Ovaria, or adhering to them by a narrow neck. These hairs differ greatly in length and color; some are only a few lines in length, some several inches, while others have been seen which measured two feet three inches. Dr. Sinnet, of Ohio, reported a case to a Committee on Ovarian Diseases of the State Medical Society, in which he opened an Ovarian cyst, finding hair eighteen inches long resembling that of the lady, and, also, in the same cyst he found five teeth in a portion of bone resembling the superior maxillary.

In almost all cases where teeth have been found, they have been implanted into the fragments of bony or cartilaginous matter, and have resembled the rudiments of maxillary bones and alveola. Meckle thinks that these accidental teeth are produced like ordinary teeth in capsules filled with a gelatinous fluid.

These serous cysts have been classed under two varieties—the *Unilocular* and *Multilocular.* They do not partake of the nature of cancer, and have no disposition to degenerate into a malignant form. Yet the Ovaria are occasionally the seat of malignant disease.

Sometimes the Ovary is affected with encephaloid disease, or is converted into a large irregular-shaped mass of cysts and tumors, the section of which presents all the character of *hematoid fungus.* This fatal affection usually runs its course with great rapidity; and soon after its commencement, the constitution of the patient is much more affected than in the organic disease already referred to. These tumors of the Ovary sometimes assume a fibrous texture, or a dense fibro-cartilaginous structure, and in rare instances contain ossific matter and chalky concretions. Encysted Dropsy, however, is the most common form of ovarian disease. It is one which has baffled the skill of the most scientific, and given rise to an operation the propriety of which has been much discussed of late, and upon which the most eminent surgeons are divided in opinion.

It has been said that this disease attacks females indiscriminately, whether they be married or single. We find, however, in collecting the statistics, that this statement is not correct, and that the married are more liable to it than the single, although the latter are frequently affected. Of 136 cases where this fact is noticed, 88 patients were married, 11 were widows, and only 37 were single—occurring in the proportion of one in 3 25-37, or 37 in 136 cases.

This result agrees with the opinion of Dr. Burns, who says that "the disease is more apt to affect those who have borne children than the unmarried." And it is opposed to that of Dr. Ashwell, who thinks that "single women are, taking a given number, and comparing them with a given number of married females, most liable to the disease.

The *age* at which the disease attacks its victims, varies considerably. There are cases, formerly supposed to be numerous, occurring before the age of twenty; and Dr. Ashwell has known one case which commenced at the early age of fourteen years, contemporaneously with menstruation; but these are rare, for out of 126 cases, only three occurred before twenty. But the most common period for its production, is when all the generative functions are in full activity, and that it is between the ages of twenty and forty years—the prime of life. And it has been ascertained that although many persons afflicted with it, arrive at a good old age, with little or nothing but their bulk to complain of, the *duration* of the disease, in the great majority, is very short. Out of 131 cases, the disease lasted only one year in 38; only two years in 25; 17 patients survived 3 years; 5 six years; 4 seven years; 3 eight years; 1 nine years, 1 ten years; 1 twenty years; 1 twenty-two years; 2 twenty-five years; and 1 thirty years. These statistics are of great importance, so far as they show the great and rapid mortality of the disease under treatment, and is an argument favorable to those who advocate the radical treatment of extirpation.

The Causes.—The causes which produce this disease, are at present only partially known. Many patients are unaware of any structural disease going on in their system until they are encumbered by its weight; some perceive an uneasiness in the side, but can not trace the disease to any cause, while others attribute it to causes impossible to admit. Of 36 cases, in which causes were assigned by the patients themselves, for the origin of the malady, 24 were connected with the reproductive process, which is certainly a most prolific source; and the fact goes far to establish the proposition already stated, that married women are more liable to the disease than the single. Of these 14 cases, five occurred directly after marriage (which is distinctly stated as a cause by the patients); nine followed parturition, some of which were observed before complete convalescence; in many cases the disease occurred after the birth of the first child, and in one case after the seventh.

After marriage and its effects, the next most frequent cause is the sudden suppression of the catamenia, and seven patients of the 36 traced their malady distinctly to it. Two cases were traced to abortion; three to exposure to cold; two to falls or blows; one to a violent fit of anger; one to an eruptive disease; and one (in a single woman) to disappointed love.

The cause supposed by Dr Denman to be the most fertile source of the malady, is the cessation of the menses. This occurred only twice in thirty-seven cases.

From these facts we draw the conclusion—as far as our numbers can be relied upon—that the production of this disease is referable in most cases to the effects of labor; that the sudden suppression of the menses is a cause next in frequency; that the excitement of marriage is next in order; and I believe disappointed affection is one of the most fertile causes in those that are unmarried.

In the majority of cases, at the commencement, the mensual process is regular as in health; but the time becomes irregular and the discharge scanty towards the close. In some cases, even where the tumor has attained an enormous size, and is obstructing the other vital functions, menstruation is unaffected.

Pregnancy has often taken place, after the formation of this disease, and parturition has been concluded without interfering with it. One case of this kind is related by Dr. Ashwell, whose advice was sought by the parents of a young girl who had ovarian disease for two years, about the propriety of marriage. He endeavored to dissuade her from her purpose, but, not taking his advice, she married and had several children without the disease producing any inconvenience. There are many cases recorded of married women having borne children after the full establishment of the disease; and in some instances, one, two and even three children were born after the tumor was of considerable size. One of the most remarkable instances of this character came under my own observation. The lady was the mother of five children, three of whom were born after the tumor had reached an enormous size.

A number of cases are reported, and well authenticated, where the patients have undergone ovariotomy, and one of the ovaria been extirpated, and yet they have subsequently enjoyed good health and borne healthy children. Some years ago, Dr. Buckner removed the right ovary involved in a tumor of twenty pounds weight, and the woman was subsequently delivered of a healthy child. In such instances, the mensual process and the power of reproduction are carried on by the healthy ovary; but when both are diseased these functions cease.

Dr. Frederick Bird, of London, reports a case in which he asserts that he extirpated both ovaria, and that menstruation was subsequently continued. This is certainly a mistake or an error in diagnosis. Otherwise, it unsettles all the teachings of our best physiologists on the subject, and we must look to some other organ than the ovaria for an explanation of the physiological changes in the uterine system, which produce the catamenial eruption. It is most probable that he removed a tumor from each side, which had its origin in the broad ligaments, but did not involve the ovaria.

The growth of an ovarian tumor is sometimes very slow, giving little inconvenience, and only becoming annoying by its bulk. It seems to proceed, for a time, independently of the general system, producing its work of destruction gradually, until at last it overwhelms its victim with alarming rapidity. I have seen a small ovarian cyst, lying dormant for a considerable time so as to throw a doubt upon the diagnosis, progress so rapidly in a few weeks, as to acquire a large size ; obstruct breathing, and severely impede the vital functions.

Many patients have lived beyond the fiftieth year of their age ; but this is rarely the case, the majority of patients, and especially where the disease is active, being carried off in a few weeks, months, or years. The usual duration of the disease is from one to two years, although 26 cases of 131 existed ten years, and one continued to exist even thirty years.

It has been stated, and upon reliable authority, that the left ovary is most liable to affection. Mr. B. Cooper holds this opinion. He says : "Of fifty cases, I find eight had some malignant disease in some other part of the body ; and that in thirteen, both ovaria were affected, and that the *left* ovary was more frequently diseased than the *right.*" This result does not correspond with the one obtained from Dr. Thomas Safford Lee's table ; for we there find, that out of 93 patients where this point was noticed (they being taken indiscriminately from the journals), the disease involved the *right* ovary in fifty cases, and the *left* in thirty-five ; while in eight of the cases, both ovaries were diseased.

In a very elaborate report on ovariotomy, read before the Ohio State Medical Society, in 1851, by the late Dr. Philip J. Buckner, more than two hundred and fifty cases are recorded, and of the instances in which the ovary affected is mentioned, the *left* was diseased in the proportion of *one* to *three* cases.

The Pathology of Ovarian Dropsy.—In considering the pathological anatomy of cystic dropsy, there are many circumstances, even at the present time, for which we shall be unable to account. The precise anatomical relations of these tumors are not clearly defined. It will be sufficient, however, to mention the more evident and prominent facts, that they may be guides in practice, and leave the disputed points to those who are more capable of entering into the discussion.

The observations, which will include the necessary practical information in relation to this disease, may be confined to three prominent particulars :

I. The various structures of cystic tumors of the abdomen.

II. Their contents.

III. Their effects they produce on the different organs contained within the abdomen.

I. The first of these divisions will contain a description of,

1. The simple cyst attached to the ovary, or broad ligaments of the uterus.

2. Enlargement of the Graafian vesicles.

3. Cysts unconnected with the ovary, found in various parts of the abdomen, and usually mistaken for ovarian dropsy.

4. Multilocular cysts.

To each of these anatomical characteristics, the attention of the reader will be briefly directed :

1. The simple cyst, which originates in the ovaries or the broad ligaments, is generally divided into two varieties, although they both possess very similar characters. The one is attached by a distinct and long pedicle, and the other is sepile. The walls are always thin, and semi-transparent, containing within their cavity a clear fluid. The peduncular variety appears to be stationary, seldom giving rise to much inconvenience ; while that which arises in the broad ligaments of the uterus may become so large as to fill the cavity of the abdomen, and present all the symptoms of ovarian dropsy, from which it can hardly be distinguished.

2. The enlargement of the Graafian vesicles is a very frequent source of cystic dropsy. We are enabled to trace this increase of size from the slight enlargement which accompanies a congestion of the ovary, to a much more considerable one, where it is evidently the seat of graver disease. And I think we are warranted in the conclusion, that many of the large ovarian tumors, where the ovary is lost, or occupies so small a portion of the sac as to be undistinguished, can be traced to the enlargement of these vesicles.

If the ovary of a woman in the prime of life be cut open and examined, there will be found under its proper covering a number of vesicles, which vary in size from a pin-head to that of a pea; there may be one largely developed, or several of a much smaller size. These, then, when they take on morbid action, may increase so much as to destroy those most contiguous to them; or one may assume a more rapid growth, and cause by its pressure the absorption of the tissue of the ovary, and thus convert it into a cyst. From this view, it is evident that ovarian dropsy very frequently arises from disease of the vesicles of Dr. Graaf, and the more its morbid anatomy is studied, the greater confirmation will this opinion receive. An additional fact, which goes far to establish this proposition, is, that the origin of ovarian dropsy, so far as it is ascertained, principally depends upon the sexual orgasm, which chiefly affects the ovaria.

3. Cysts are not unfrequently developed in other parts of the abdomen, unconnected with the uterus and its appendages, producing the same or similar symptoms, requiring the same treatment, and undergoing the same changes, as those which have their origin in the ovaries. This is illustrated by a case which occurred under the care of Dr. A. T. Thompson, of University College Hospital. It partook of all the symptoms of ovarian dropsy: the patient was tapped forty-eight times, from which operation 177 gallons of fluid were discharged. The swelling commenced in the form of a tumor in the lower part of the abdomen toward the right side, which gradually extended to the left, and increased to such an extent as to interfere with digestion and respiration. On examination, after death, the tumor was found to have arisen in the *omentum*, close by the pancreas, and was attached by a long, thin portion to the uterus, but it was entirely unconnected with the ovaries.

A very remarkable case of this kind is given by Dr. T. S. Lee. Mrs. ——, 50 years of age, married, had been laboring under a tumor of the abdomen for twenty-five years. She had had one child previous to its appearance, and three children since; she suffered in nothing from the disease, except its bulk, and up to the last, was able to amuse herself with household duties. The tumor was of enormous size, disturbing the breathing, and at last terminated fatally.

A *sectio cadaveris* revealed the following facts: The cavity of the abdomen was almost entirely filled with an enormous tumor, which pushed up the viscera to the right side, and compressed the spleen posteriorly. It was found to have commenced on the left side, just under the pancreas, *but below the peritoneum*, so that it rested upon the posterior muscular walls of the abdomen. A narrow pedicle, six inches long, of the size of a quill, connected it with the uterus. It had, also, formed connections with the other viscera of the abdomen. The cyst itself contained two pailsful of turbid, whitish-colored fluid, with an immense number of balls of hair, mixed with fat, and calcareous matter; no hairs were observed attached to the cyst, but the balls of hair, fat, and osseous deposit were as large as the closed hand. On the left side of the cyst, attached to the walls, was a mass of bone and teeth, etc., strongly resembling an imperfect fetus. This body was about four inches long, and covered by a membrane resembling the true skin, but closely connected with the sac. It presented at the upper portion an opening divided into two parts, like imperfect nostrils, immediately under which was a large bone, like the lower jaw, filled with teeth; on each side of this part projected a small appendage resembling the ear; below this mass were two long appendages, like abortive arms; the right one smaller, and composed of skin, at the end of which were a few hairs; the left was larger, still more closely resembling the arm, and apparently jointed at the shoulder and elbow; it contained one strong bone like the humerus, and two small bones for the forearm, but the lower end of these terminated the limb. At the lower extremity of the body of this mass was a large projecting bone, also jointed. This approached the form of a femur, at the lower extremity of which was an irregular deposit of bone.

This case, during life, presented all the appearances of ovarian dropsy. After death, it was found to possess masses of hair, and a body analogous to an imperfect fetus; and yet the cyst was located *beneath the peritoneum*, and consequently entirely detached from the uterine organs.

It has long been supposed that such productions are the result of extra-uterine conceptions; but this, at least, is not universally the case, as the foregoing post-mortem examination clearly demonstrates.

4. *Multilocular Cysts.* The symptoms to which this species of cyst gives rise differ materially from those produced by the varieties we have already noticed. Instead of distinct and clear fluctuation, we find this symptom very obscure, and only distinct in particular positions. This arises from the tumor being divided into a number of separate cavities, with walls sufficiently thick and tense to prevent the fluctuation of one cyst affecting the fluid of another. This is found to be the case even where the sac is removed from the body.

The form of the tumor, where it is multilocular, is irregular, from the projection of secondary and tertiary cysts into its cavity and beyond its walls. Such portions feel fleshy, and when the hand is applied over them, fluctuation is perceived much less distinctly. The growth of these tumors is much more rapid than the other forms, its effects on the constitution greater, and its final results more certainly fatal. From the first appearance of the disease, a deep-seated pain is felt in the groins, and examination reveals a hard tumor, which may be mistaken for a tumor of the uterus or ovary. This may gradually enlarge, or become stationary for a time, but then suddenly increase, until it fills the entire cavity of the abdomen, pressing upon the diaphragm, affecting all the vital functions, producing dispnœa, vomiting, œdema, and death.

The mode of the formation of these tumors, has been a source of great discussion among pathologists. Some believe that this particular variety is the product of hydatids; others, that it is enlargement of the cellular tissue; while others think that the growth is merely adventitious. This last opinion is advanced by Dr. Hodgkin, and is now generally received as correct. A glance at his principal arguments will be interesting: *(Hodgkin on Serous Membrane).*

Speaking of the adventitious serous membrane, he says, "that the adventitious serous membranes, like those existing naturally in the body, form complete shut cavities. As far as in our power to ascertain, they are wholly, or at least with very few exceptions, the result of an entire new formation, dependent on some anomally in the function of nutrition, but with regard to the precise nature of which we are completely n the dark." He goes on to divide serous cysts into two distinct classes; the one, where they are simple, and, for the most part, solitary, and containing only one cavity; the other, where the sac possesses the remarkable property of giving origin to new growths having the same character as itself. The latter is the multilocular form, and will now engage our attention.

"In this form," says Dr. Hodgkin, "we observe on the interior surface of the principal cyst, elevations more or less rounded, and of various sizes, projecting into the interior of the cavity, and covered by a membrane which is continuous with the lining of the principal sac. On making an incision into these tumors, we find that they also consist of cysts of a secondary order, filled by a secretion, often serous, but almost as frequently mucous. It is not, however, merely by these secretions that these cysts are filled; on looking more minutely on the interior of these cysts, there grows a cluster of other or tertiary cysts, upon which is reflected the lining membrane of the cyst in which they are contained. Cysts of a secondary order, not unfrequently afford as complete specimens of a reflected serous membrane, as either the pericardium or tunica vaginalis: the lining membrane of the containing cyst corresponding to the reflected portion, as that covering the contained bunch of cysts, does to the closed portion. The proportiou which the contained cysts bear to the cavity of the membrane reflected over them, is extremely various. Sometimes the fluid, especially where it is of a serous character, nearly fills the containing cysts, while the bunch of cysts is of a very inconsiderable size. At other times, the superior cyst is almost filled with those of an inferior order; in which case we may generally find that the nodules, or tuberous elevations, which we may have observed on the exterior of the containing cyst, are occasioned by the unequal development of the contained cysts; for those which have grown most rapidly, and have obtained the largest size, forcibly dilating that portion of the cyst which is reflected over them, produce a kind of hernia of that part. It sometimes happens that the distension, occasioned by the growth of the contained cyst, is sufficient not only to distend the even surface of the containing cyst, but actually to produce a rupture, which admits both of the escape of its fluid contents, and of the uncompressed growth of the secondary or tertiary cysts which took their origin from its surface.

The cysts which I have been describing, as found on the internal surface of the first formed cysts, at times pour out a part of their contents into the interior of the large or parent cyst, either in consequence of an extensive rupture produced by the development of a contained order of cysts, as I have before described, or by small apertures, which likewise appear to be the result of distension. In both these cases, but especially the latter, the open cysts bear a considerable resemblance to mucous follicles on a large scale, and appear to be the principal sources of the very copious and rapidly-produced secretion which is a characteristic feature in many cases of ovarian dropsy. This mucous bears a very close resemblance to that furnished by the glands of Naboth, and it is frequently so viscid that it passes with difficulty through the canula. The membranes of which these cysts, whether of the secondary or tertiary order, are formed, are liable to inflammation. The product of this inflammation, like that which takes place in the serous membranes, naturally belonging to the body, may be either of the plastic or unorganizable kind. In the former case, it leads to the formation of adhesions between the close portion of the membrane, or that which constitutes the cluster of cysts, and that portion which is reflected over them, forming the varieties of the containing cysts.

When the product of the inflammation is of the unorganizable kind, we find a secretion more or less puriform in its character. This secretion is found sometimes confined to one or more of the secondary cysts; at other times, it finds its way of escape into the interior of the principal cyst, and thus contributes to the variety in the appearance presented by the fluids, drawn off in the operation of paracentesis for the relief of ovarian dropsy. But the puriform secretion may proceed from the abrasion of the principal cyst."

This, then, I conceive, is the most plausible theory of the primary formation of multilocular cysts. But there are other peculiarities which come under our notice, and deserve mention.

*The size* of these tumors varies greatly, from the small pedunculated cyst, to one which will measure four feet, or more, in circumference. No definite extent can be given to them, and the simple and complicated equally attain an enormous size. Their tendency is to increase until arrested by some pressure they can not overcome. Their growth is very rapid usually; the great majority of cases, as before observed, terminating fatally within two years.

*The walls* of these tumors are aponurotic, varying greatly in thickness: some are very thin, while others are thick and fleshy. Externally, they are generally smooth and shining, when undetached; internally, they present a variety of appearance. In some the lining membrane is rough and granular, and often thrown into the folds of different forms, from the diminution of the cyst by tapping or rupture. Sometimes a great portion of the cystic walls is very thick, and has, apparently, an outer and inner layer, with the intervening space filled with small cells; the whole mass, when cut, has the appearance of honeycomb, or a piece of sponge. Some of these cells are distinct, others communicate with those contiguous to them. I have seen the walls of one of these cysts two inches in thickness. Occasionally the cysts on the internal surface of the principal one, are pedunculated, presenting the appearance of a cauliflower, rough, granular, hard, and filled tensely with fluid. These masses may occupy only detached portions of the cyst, or entirely fill its cavity. Fibrous tumors may, also, project from the inner surface; and bony matter is frequently deposited within the walls, either in distinct patches, more or less extensive; or it may be deposited throughout the whole extent of the parietes of the cyst.

Hydatids have been frequently found in ovarian cysts. They are distinct, globular bodies, which support their own life, are unattached, floating in the fluid of the cyst, and appear to be foreign to it.

Ovarian tumors are very freely supplied with blood. Blood-vessels can be seen ramifying over them, and the principal cyst is sometimes so vascular as to cause its internal surface to be quite injected. Large blood-vessels are frequently seen running in all directions, both between the various cysts and externally on the tumor; and when present, add materially to the danger of paracentesis. I have seen vessels as large as the little finger ramifying in all directions over a tumor of this description; and in one case, a large vessel ran below the umbilicus, between the cyst and abdominal walls. Many are the instances which may now be cited, of death from hemorrhage as a consequence of extirpation of these tumors. The pedicle is often so largely and freely supplied with blood-vessels, that no ligature is sufficient to secure it.

The adhesions these bodies form with the neighboring viscera, will be fully considered, when we come to treat of ovariotomy as a means of cure.

II. *The contents of ovarian cysts.* These may be divided into *fluid* and *solid.* Although we have briefly enumerated the varieties, and glanced at their character, it may not be uninstructive to consider them a little more in detail.

In the first class (fluid) we find great varieties. In the simple cyst there is generally to be found a transparent, straw-colored fluid, which is highly albuminous, coagulating on the application of heat or nitric acid. When such fluid is discharged, and entirely empties the tumor, the probability is that the cyst is simple, and the disease, at that time, benign; but if the tumor be not emptied, we can not draw such a conclusion, for this fluid may be only the contents of one of a multilocular cyst.

The next secretion most common in this disease, is that of a thick, glary, gelatinous semi-fluid, varying in consistence from that of a thick cream to almost solid matter. Frequently a fluid-like coffee is discharged. Under the microscope, it is found to contain blood corpuscles, in their perfect state, some with their capsule destroyed, and also small detached pieces of capsule without a definite form.

A light brown fluid may be drawn off, and towards the close of the operation, distinct white masses present themselves of various forms, preventing the exit of the remaining fluid by closing the canula. A fluid of this kind was found to be of specific gravity 1.025, and it became nearly solid on the application of heat or nitric acid; but it did not coagulate spontaneously. The solid matter under the microscope appeared to consist of granules, and the mass appeared to be fibrous. There were, also, similar globules contained in the fluid, with numerous blood-disks. Pus is often effused into these sacs after an inflammatory attack, and large quantities are discharged. Sometimes we meet with a fluid of an olive green color, containing a number of shining crystals: these are found by the microscope to be cholesterine.

Dr. Rees has given an analysis of five specimens of fluid taken from ovarian dropsies, and has compared them with the constitution of the blood, and finds in them an excess of water and extractives, but a deficiency of albumen. He says "it will be seen by comparing these analyses with that of the serum of the blood, that in every specimen there is a considerable excess of water and extractives, and a deficiency of albumen. As all these fluids were of that mucoid tenacious character, so well known to those who are in the habit of examining the cyst of ovarian dropsy, I am inclined to conclude that this peculiarity of appearance is attributable to the presence of a large portion of extractives, particularly the albumen combined with soda, which opinion is confirmed by the experiments of Dr. Babington, who has succeeded in forming a mucoid fluid by the addition of alkalies to albuminous fluids or secretions.'' In the preparation the salts are in excess in proportion to the albumen.

"My reason," says Dr. Rees, "for regarding the salts in relation to the solid matter, is, that the peculiar mucous character of the liquors is owing to the nature of the solid ingredient, and quite independent of any peculiar portion of water, as might at first be supposed. Again, the alkaline salts obtained from the ovarian fluids, differ from those of the blood in not containing any phosphate which can be recognized, even as a trace, in the quantity of solid matter obtained from two hundred grains; experiments made on large quantities for the express purpose of detecting an alkaloid phosphate, showed a trace only.

From these experiments, then, we find that the fluids taken from ovarian cysts, when compared with the blood, contain *less* albumen than that fluid, although *more* than it, in combination with soda; and the peculiar mucoid character of such fluids depends upon an excess of extractives, particularly albumen combined with soda.

All the varieties of fluid may be contained in one ovarian tumor, as is often demonstrated by paracentesis. At first a pale yellow fluid may be evacuated, followed by a black and tenacious one, succeeded again by pus.

This depends upon the bursting of secondary and tertiary cysts into the principal one, or inflammatory action resulting in ulceration. After death we frequently find these various productions contained in neighboring cysts. Mr. Howard, in the Medical Gazette, 1852, describes a tumor of this sort, he says: "The tumor was composed of an immense number of cysts of all sizes; their contents were very various—in some, the matter was colorless serum, in others it was yellow, in others as dark as coffee, and in others it was bloody. The consistence of these fluids was as various as their color."

We shall next give a brief description of the *solid substances* contained in ovarian tumors. Fat, hair, bone and teeth, as we are already informed, are the solid substances most usually met with. Formerly these productions were always attributed to *conception*, but subsequent observations have distinctly proved that the generative faculties have nothing to do with their formation. And the facts produced in proof of this statement arise, 1st, From the knowledge that these productions have been observed in virgins, and those too young for copulation. 2d, They have been discovered in other parts of the body, unconnected with the uterus or its appendages. And 3d, they have been found in the male sex.—*Vide. Baillie's Morbid Anatomy.*

1. Dr. Baillie has furnished us proof of the first fact, and has related a case where fat and hair were found in the ovaria of a little girl thirteen years of age. A little girl about thirteen years old was brought into the dissecting room, and the blood vessels were injected. The right ovarium was swelled to a size larger than a hen's egg. It was filled with a peculiar sort of fat and hair; at one place there were two long excrescences from the capsule containing this fat, which a good deal resembled teeth. The uterus was as small as at birth, and when opened, exhibited the common appearances. The girl had an entire hymen and the pubis was without hair. Such cases have been considered as impregnations, but in this case, the state of the uterus, the age and the hymen rendered such a supposition groundless.

2. These substances are found in tumors altogether unconnected with the generative organs. We have already referred to a case where the cyst contained in its walls a body resembling an abortive fœtus, which was entirely unconnected with the ovaries, and was situated *under the peritoneum*, and lying on the muscular tissue of the posterior walls of the abdomen. In that case there were two large portions of bone, regularly set with teeth, corresponding to and representing two portions of the lower jaw, and other large bones were seen. The cyst which contained these bodies, and from which they grew, was of enormous size, and contained large quantities of fat mixed with hair. The disease was of twenty-five years standing. The uterus and ovaries were healthy and unconnected with the tumor.

Tumors containing these substances are frequently found in other cavities besides the abdomen. Dr. Gordon, of the London Hospital, has met with a tumor in the anterior mediastinum, containing an osseous structure resembling a portion of the superior maxillary bone, some hair and teeth. And Sir B. Brodie has found some well formed teeth in the bladder.

3. These formations have, also, been found in animals, and in the male sex. Professor Coleman has described a tumor found in the abdomen of a gelding, in which two molar teeth of the horse, possessing the regular arrangement of bony matter and enamel, were attached; also, an incisor attached to a portion of a bone resembling the jaw, and a quantity of hair and fat in a separate cyst.

Dr. F. H. Ramsbotham, also, states in his lectures that Ruysch possessed a tumor in his collection which consisted of teeth and hair, that had been taken after death from a cyst found in the coats of a man's stomach; besides a jaw with well formed teeth which had been taken from the bladder.

Duvernay saw a tumor extirpated from the scrotum, containing fleshy matter and bones.

Dupuytren related to the Medical Society of Paris the history of a tumor found in the abdomen of a boy, containing a mass of hair, and a fetus nearly ossified.

And in the first volume of the *Med. Chir. Transactions*, page 236, is a description, by Mr. George W. Young, of a fetus distinctly recognized in a cyst, in the abdomen of a boy (John Hare) about a year and a half old.

These cases are quite sufficient to prove that such productions are not the result of the procreative function—that they are not extra-uterine conceptions; but, more probably, that they are either the production of the cyst itself, or of the confusion of two separate ova at the time of impregnation. No doubt the latter supposition may account for some of these anomalous products; but we think sufficient stress has not been laid on the secreting powers of the cyst itself. For instance: we have already seen that it is no uncommon thing for a cyst to secrete bone; we have quoted instances where the sac itself has been converted into bone; and we have seen bone discharged from an ovarian tumor, during life. Besides, hair, also, can be produced by the cyst itself. In the museum of St. Bartholomew's Hospital, there is an ovarian sac, the inner surface of which has taken on a peculiar action, and has produced a membrane like the scalp, which is covered with hairs, they having a distinct bulb, and growing in the same manner as on the external surface of the body. Dr. Carswell, also, gives a beautiful drawing of an ovarian cyst, from a portion of which grew a considerable number of long hairs; some hairs were detached, and had formed themselves into balls of various sizes. No hairs grew from any other portion of the cyst, and they possessed bulbs. We thus find, that the sac contains the power, not only of producing hair, similar, in every respect, to the natural hair of the body, but of bone itself, which becomes detached like the hair, and is found in variously shaped masses, in the cavity of the cyst.

Dr. Ashwell supposes that these products have their origin from disappointed sexual appetite; the power of production being present in the female, but the ovum not receiving the vivifying stimulus of the male semen, an imperfect development is the result. But if we, for one moment, consider the foregoing cases, and reflect that they are found in all parts of the body, and in parts entirely unconnected with the generative system, and even in the male sex, this supposition of Dr. Ashwell must be erroneous.

III. We come now to consider, in the third place, the effects produced on the abdominal viscera by ovarian cysts.

In the early stages, these tumors more particularly affect by pressure the organs contained within the pelvis, causing retention of urine, and constipation; producing, also, various changes in the position of the uterus. At a later stage, the results of pressure are felt in a more serious degree: the stomach is affected; the chest is unable to perform its functions; the kidneys are pressed upon; suppression of urine takes place; and sometimes, by the bursting of the sac, the peritoneum becomes inflamed.

There is great tendency in these tumors to produce ulceration in neighboring organs. The colon, throughout its extent, is subject to its ravages: many cases are recorded to illustrate this fact. The bladder has been perforated by the pressure of these tumors. Dr. O. Heming relates a case, in the translation of M. Boivin's and Duge's work, where "the bladder was opened by ulceration, and for a long time allowed hair to pass with the urine; at last a body was extracted from the bladder, as large as a hen's egg, presenting at one of the extremities a shred of skin containing hairs, and a bone in which was partially fixed a kind of tooth resembling a small molar. The communication of the cyst with the bladder was ascertained by the finger passed into the urethra. The person is said to have recovered."

These tumors, when small, and existing with pregnancy, give great trouble when parturition occurs, and frequently endanger the life of both mother and child.

The late Dr. Buckner, of Cincinnati, whose manuscript notes upon this subject I am permitted to examine, has given the following case: "I witnessed a post-mortem examination of a female in this city, who died suddenly from an obscure disease of the uterus, attended by profuse periodical hemorrhage. The abdomen was considerably enlarged by a solid tumor in the hypogastric region, extending as high as the umbilicus. In the left iliac region was another tumor, soft, and more elastic than the other. The periodic hemorrhage was preceded and accompanied by severe pains, resembling those of labor. During life the lady was attended by several medical gentlemen, of respectable acquirements, and several others, of great experience, were called in consultation. Yet none were fully able to diagnosticate the case. It was treated for dysmenorrhea; it was pronounced ovarian disease; some called it polypus of the uterus; while others thought it malignant disease of the uterus, complicated with ovarian dropsy. The *post obit* revealed the existence of ovarian cyst, about the size of a full grown fetus, filled with a transparent fluid. The uterus was enlarged to about the same size. On cutting it open, the walls were found thick and indurated, and a large bloody fungus projected from the inner surface of the fundus, through the *os tincæ,* into the vagina. Extensive adhesions existed to the rectum, bladder, and peritoneum adjacent. In the posterior wall and fundus of the uterus, was found extensive ulceration within the compass of the adhesions with the rectum. This abscess had ruptured, preceding death, and discharged a large quantity of pus into the abdominal cavity; thus inducing death from actual inflammation in the peritoneum and intestines."—*Cincinnati Medical Observer.*

(TO BE CONTINUED.)

